# Core-Shell Fiber-Based 2D Woven Triboelectric Nanogenerator for Effective Motion Energy Harvesting

**DOI:** 10.1186/s11671-019-3144-2

**Published:** 2019-09-11

**Authors:** Jinmei Liu, Long Gu, Nuanyang Cui, Suo Bai, Shuhai Liu, Qi Xu, Yong Qin, Rusen Yang, Feng Zhou

**Affiliations:** 1 Institute of Nanoscience and Nanotechnology, Lanzhou University, Lanzhou, 730000 China; 2 School of Advanced Materials and Nanotechnology, Xidian University, Xi’an, 710071 China; 3 State Key Laboratory of Solid Lubrication, Lanzhou Institute of Chemical Physics, Chinese Academy of Sciences, Lanzhou, 730000 China

**Keywords:** Wearable power source, Triboelectric nanogenerator, Energy harvesting, Mechanical durability, Power fabric

## Abstract

Personal electronic devices have a general development trend of miniaturization, functionality, and wearability. Their wireless, sustainable, and independent operation is critically important, which requests new power technologies that can harvest the ambient environmental energy. Here, we report a new kind of 2D woven wearable triboelectric nanogenerator (2DW-WTNG) composed of core-shell fibers via the twisting process and weaving process in the textile manufacture. The 2DW-WTNG can convert the body motion energy into electricity with an output current of 575 nA and an output voltage of 6.35 V. At an external load of 50 MΩ, it generated a maximum power density of 2.33 mW/m^2^. Electricity can be produced from the 2DW-WTNG driven in arbitrary in-plane directions. A tiny displacement of 0.4 mm can drive the 2DW-WTNG, which verified its capability to harvest energy from small human movement. The robust 2DW-WTNG can work continuously for 12 h without obvious performance degradation.

**Electronic supplementary material:**

The online version of this article (10.1186/s11671-019-3144-2) contains supplementary material, which is available to authorized users.

## Introduction

With the fast development of the electronic technology, various portable, wearable, and even implantable personal electronic devices have been invented to make our daily life better [1–11]. Nevertheless, due to the inherent limitations of traditional batteries, such as limited capacity, short lifetime, maintenance difficulty, and environmental hazards [12–14], the increased production and wide application of personal electronic devices make pressing demands for new power supply [15–19]. It is urgently needed to develop new power sources that enable electronic devices working sustainably and timely. Body motion energy from human daily activities widely exists no matter what you do and where you are [20]. If such mechanical energy can be effectively harvested and converted into electricity, it may power personal electronic devices whenever and wherever needed.

As a new type of power-generating device to convert mechanical energy into electricity [21–24], triboelectric nanogenerators are based on the coupling of the contact electrification effect and the electrostatic induction [25–30]. They have been successfully demonstrated as sustainable power sources for portable electronics, sensors, environmental monitors, and so forth [31–44]. Among them, wearable triboelectric nanogenerator (WTNG) has been designed to convert the body motion energy from human activities into electricity [45–47]. The current WTNGs can be divided into two categories by whether a substrate is used. Most WTNGs belong to the first category and have their electrode and frictional layer coated on a flexible substrate, such as a textile fiber or a piece of fabric [48–53]. They possess good softness, flexibility, and light-weight. Nevertheless, the adhesion between the loaded electrode and the substrates is poor, which greatly reduces their durability and usability, and further makes these WTNGs unavailable for long-term usage. The second category of WTNG does not rely on extra substrate, and their building materials are directly used as the frictional layers with electrodes. A WTNG based on nylon cloth and polyester cloth skillfully avoided the adhesion issue from the substrate [54]. Later, a kind of WTNG with stainless-steel conductive thread as the electrode and silicon rubber and PDMS as the frictional layer materials were developed [55–57]. However, these WTNGs either do not have long-term robustness or have a quite complicated fabrication process which can be used in large scale fabrication.

In this work, we fabricated a new kind of 2D woven wearable triboelectric nanogenerator (2DW-WTNG) with merits of robustness and continuous production process which is well suited for large scale production. A 2DW-WTNG with a size of 1.5 × 1.5 cm^2^ generated an output voltage and output current of 6.35 V and 575 nA, respectively. Connected to an external load of 50 MΩ, it generates a maximum power density of 2.33 mW/m^2^. After connected with a rectifying bridge, the 2DW-WTNG instantaneously powered a commercial light-emitting diode (LED) without any energy storage process. It was also used to charge a 0.47 mF capacitor from 0 V to 1.84 V in 1 min. Furthermore, it showed good sensitivity to external motions down to a displacement of 0.4 mm, good adaptability to work along arbitrary in-plane directions and in different working modes, and good robustness to work continuously for 12 h without degradation.

## Methods

### Fabrication of the Nylon/Copper Core-Shell Composite Conductive Fiber and Polyester/Steel Core-Shell Composite Conductive Fiber

**Fig. 1:**
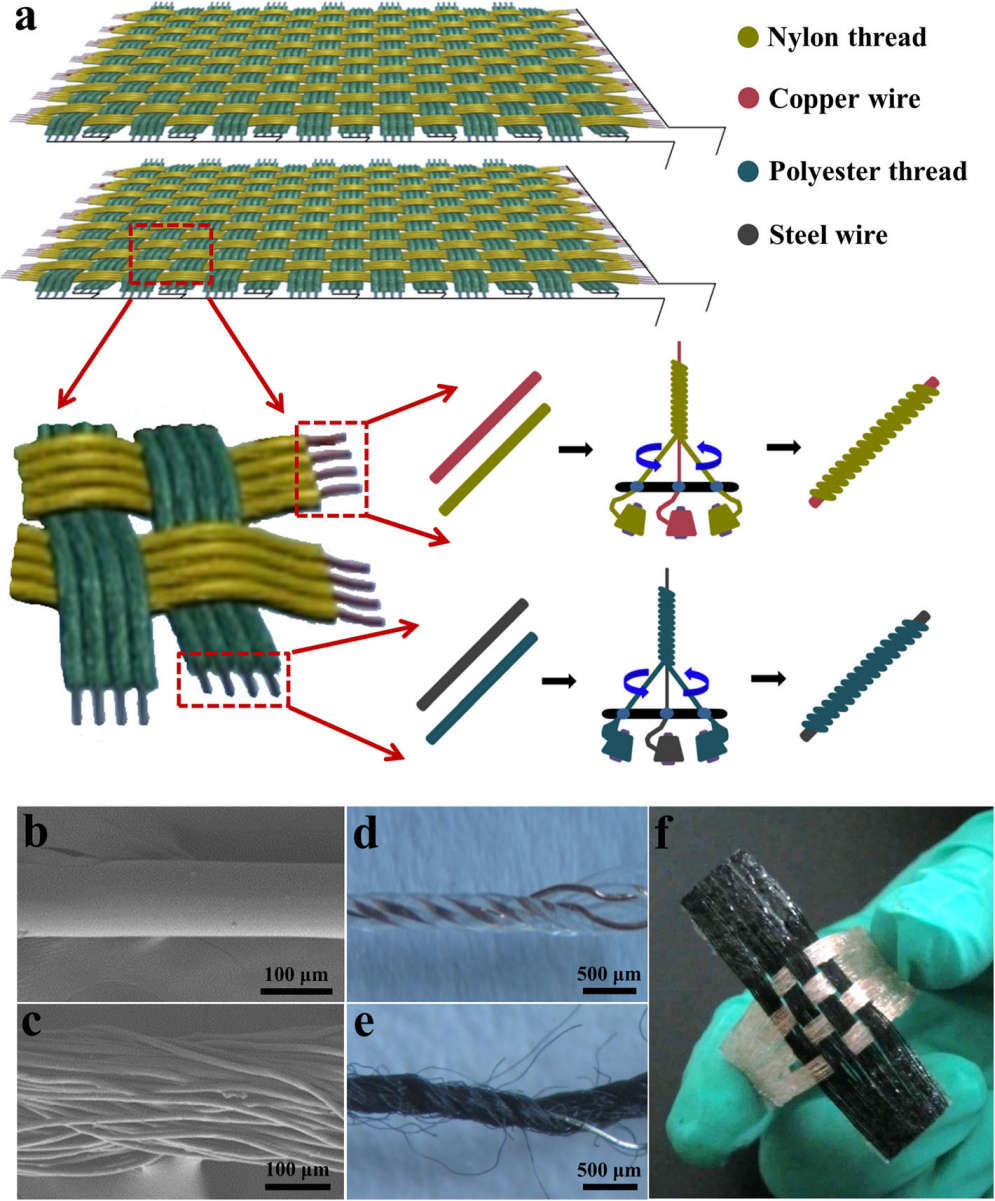
Fabrication and structure of the 2DW-WTNG. **a** Schematic diagram illustrating the fabrication process. SEM images of the nylon thread (**b**) and the polyester thread (**c**), respectively. Optical images of the nylon thread coated copper wire (**d**) and the polyester thread coated steel wire (**e**), respectively. **f** Optical images of the 2DW-WTNG

The source materials for the nylon/copper composite fiber are daily sewing nylon thread (110 μm in diameter) and the enameled copper wire (60 μm in diameter). The source materials for the polyester/steel composite fiber are daily sewing polyester thread (200 μm in diameter) and the steel wire (60 μm in diameter). Two kinds of polymer/metal composite fibers were prepared using a homemade rotating support as shown in Fig. 1. For the preparation of the nylon/copper composite fiber, the enameled copper wire was first fastened at the middle of the rotating support and then two nylon threads were fixed at two ends of the rotating support. Next, the top of these three wires was held together and hung up. Finally, starting from the rotating support, nylon threads were twined and wrapped around the middle copper wire, and the nylon/copper composite fiber with core-shell structure (380 μm in diameter) was formed. The preparation for the polyester/steel composite fiber was similar to that of the nylon/copper composite fiber, in which the enameled copper wire was replaced with the steel wire and the nylon thread was replaced with the polyester thread. The core-shell structure was achieved with a steel wire tightly coated with polyester thread (385 μm in diameter). Here, different metal wires were chosen to balance the stability and output performance of the 2D-WTNG. Compared with the steel, the copper wire was covered with a thin insulating layer, which was used to avoid the short circuit during the 2D-WTNG’s working process. If the steel was selected to be the core electrode for both fibers, friction and abrasion might happen after a long time working, in which short circuit may occur between the positive electrode and the negative electrode. This will decrease the 2D-WTNG’s stability. If the copper was selected to be the core electrode for both fibers, the electrostatic induction effect would be weakened by the insulating layer on the surface of the copper wire, which will reduce the 2D-WTNG’s performance. This preparation process for the polymer/metal composite fiber imitates the model of the twist tuo, which is a simple tool in hand twisting thread. Using this method, the polymer/metal composite fiber can be put into mass production using a twisting machine in the factory.

### Fabrication of the 2DW-WTNG

The prepared nylon/copper composite conductive fiber and polyester/steel composite conductive fiber were integrated into fabric by the general knitting technique. Nine nylon/copper composite fibers were put together side by side as a group, and nine polyester/steel composite fibers were put together side by side as a group. Two groups of nylon/copper composite fibers and two groups of polyester/steel composite fibers were knitted into the WTNG with 2D woven structure. The upper and lower parts of 2DW-WTNG have a size of 15 mm × 15 mm (composed of 36 composite fibers) and 38 mm × 38 mm (composed of 90 composite fibers), respectively. Their grating width was about 7 mm as shown in Fig. 1. Here, the grating width is determined by the diameter of the composite fiber and the number of the composite fiber used in one group; thus, the grating width can be conveniently adjusted by increasing or decreasing the composite fiber number in one group. This woven process can be done on loom in the factory when needing massive production.

### Measurements of the 2DW-WTNG

The 2DW-WTNG with an effective size of 15 mm × 15 mm and a grating width of 7 mm was tested by periodically moving back and forth. In the measurements, the lower 2DW-WTNG was fixed on a mechanical stage that was tightly mounted on the experimental table, and the upper 2DW-WTNG was put on top of the lower 2DW-WTNG and connected with a linear motor. Driven by the linear motor with controlled sliding displacement and velocity, the upper 2DW-WTNG makes a reciprocating linear motion in the range of the lower one at a direction parallel to the surface of the device, which keeps the effective contact area unchanged all the time.

## Results and Discussion

By using the twisting technology in the textile manufacture, a core-shell structural composite fiber was fabricated with metal wire as central electrode and sewing threads as outer frictional layers. The 2DW-WTNG was further fabricated by weaving two kinds of core-shell structured composite fibers through an orthotropic weaving process. Figure 1a shows the structural illustration of the 2DW-WTNG with two same parts. In each part of the 2DW-WTNG, nylon/copper composite fibers arranged in one direction were collected as one electrode, and polyester/steel composite fibers arranged in the other direction were collected as the other electrode. Two kinds of composite fibers were prepared using a homemade rotating setup working like a twisting machine in factory. Scanning electron microscope (SEM) images shown in Figs. 1a and c reveal the surface appearance of the initial nylon thread with a diameter of 110 μm and polyester thread with a diameter of 200 μm, respectively. Figures 1d and e are the optical images of the prepared nylon/copper composite fiber and polyester/steel composite fiber, respectively, from which the core-shell structure can be observed distinctly. Figure 1f exhibits an optical image of the final 2DW-WTNG made of four groups of nylon/copper composite fibers and four groups of polyester/steel composite fibers. With the woven structure in length and breadth, it resembles a piece of common cloth, and the detailed fabrication process is illustrated in the “Methods” section.

**Fig. 2:**
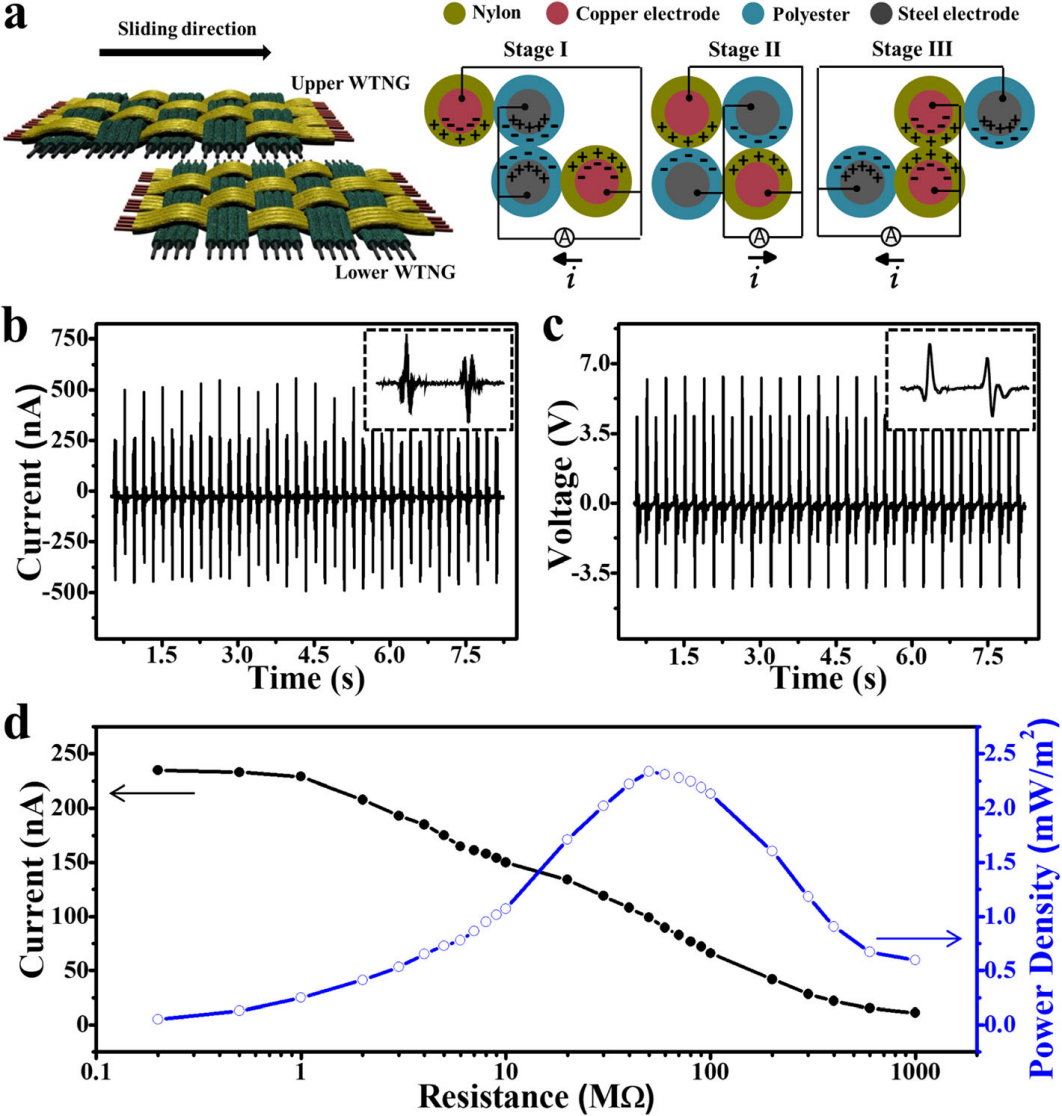
Power generation characteristics of the 2DW-WTNG. **a** Overall process of the electricity generation mechanism. **b** Output current and **c** output voltage of the 2DW-WTNG. The inserts in **b** and **c** are enlarged views of the output current and output voltage. **d** Current (solid circle) and power density (open circle) of the 2DW-WTNG with different load resistances

The power generation performance of the 2DW-WTNG is studied. As shown in Fig. 2a, the upper part and the lower part of the 2DW-WTNG were fixed face to face, and the upper part can slide rightward and leftward against the lower part. Once relative sliding occurred between the upper part and the lower part, the contact surfaces rubbed with each other. Since polyester is more triboelectrically negative than nylon according to the triboelectric series, electrons are injected from nylon into polyester, producing positive triboelectric charges on the nylon surface and negative charges on the polyester surface. When the upper part slid rightward and the contact surface slid into a misaligned position as shown in stage I, net electric field arose as a result of uncompensated triboelectric charges in the misaligned regions, driving free electrons from the electrode in polyester moving to the electrode in nylon until the electric field was screened by the induced charges on electrodes. When the upper part continued sliding rightward, the contact surface came into an aligned position and the triboelectric charges of opposite signs were completely balanced, leading to a back flow of the induced free electrons (stage II). As the rightward sliding went on, the contact surface was brought back towards the misaligned position, and free electrons were driven from the electrode in polyester to the electrode in nylon as shown in stage III. Consequently, a cycle of the electricity generation process for the 2DW-WTNG was completed. Benefited from the grating design with interphase structure between nylon/copper composite fiber and polyester/steel composite fiber, charge alternately transferred between two electrodes during this process. Experimentally, the initial contact situation depends on how the upper part and lower part are placed. However, it will not influence the output of the 2DW-WTNG. Owing to the same grating structure, the initial contact situation makes no difference in the output peak value but changes the direction of the output peak. If the initial contact situation is positive to positive, the contact situation will first get into positive to negative and then into positive to positive with a positive output peak followed by a negative output peak. In contrast, if the initial contact situation is positive to negative, the contact situation will first get into positive to positive and then into positive to negative with a negative output peak followed by a positive output peak.

A 2DW-WTNG with a size of 15 mm × 15 mm and the grating width of 7 mm was tested by periodically moving back and forth. The detailed measurement method is illustrated in the “Methods” section. With a sliding displacement of 8 mm and a sliding speed of 0.15 m/s, the 2DW-WTNG produced a continuous alternating current (AC) output at a maximum amplitude of 575 nA at a constant frequency of 2.7 Hz (Fig. 2b). The output voltage reached 6.3 V at the same frequency as the output current (Fig. 2c). The enlarged view of the output current and output voltage in one working cycle are inserted in Figs. 2b and c, respectively. In one working cycle, there are two wave packets, one representing the one-way sliding rightward and the other representing the one-way sliding leftward. And there are two positive pulses and two negative pulses in each wave packet. This result is in accordance with the structure of device containing four groups of nylon/copper composite fibers and four groups of polyester/steel composite fibers as shown in Fig. 1f, which further verifies that the output in sliding mode is closely connected with the grating width and grating number in the device.

As a power source in practical, the 2DW-WTNG needs to be connected with external loads. Resistors were used to investigate the reliance of the output electric power on the external load. Figure 2d shows the instantaneous current and the instantaneous output power density versus the external load resistance. The instantaneous output power density was calculated as the ratio of the instantaneous output power (*I*^2^*R*) and the area of the device. It was found that the instantaneous current dropped with the increase of load resistance owing to the ohmic loss. The instantaneous output power density increased at low resistances and reached a maximum value of 2.33 mW/m^2^ at the load resistance of 50 MΩ, and then decreased at higher resistance. This result indicates the potential of the 2DW-WTNG to be a power supply for some personal electronic devices, especially for those with a load resistance about dozens of megohm.

**Fig. 3:**
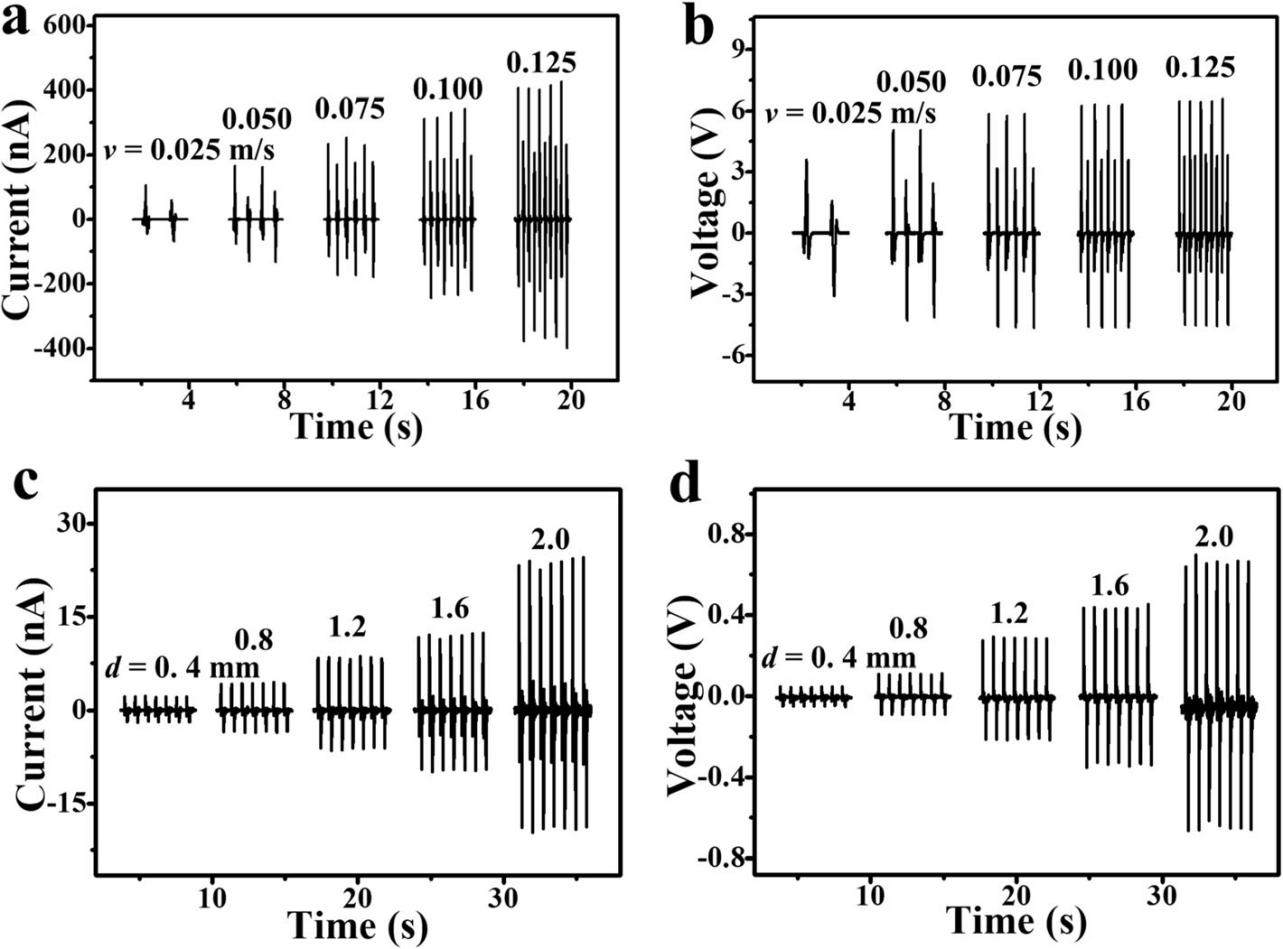
Power generation performance of the 2DW-WTNG under different relative sliding speeds and relative sliding displacements. **a** Output current and **b** output voltage of the 2DW-WTNG varied with the sliding speeds of 0.025 m/s, 0.050 m/s, 0.075 m/s, 0.100 m/s, and 0.125 m/s at a given sliding displacement of 8 mm. **c** Output current and **d** output voltage of the 2DW-WTNG varied with the sliding displacements of 0.4 mm, 0.8 mm, 1.2 mm, 1.6 mm, and 2.0 mm at a given sliding speed of 0.100 m/s

The output performance of the 2DW-WTNG in sliding driven mode greatly relies on the separation rate of triboelectric charge. To study this deeply, the output performance of the 2DW-WTNG with a size of 15 mm × 15 mm and a grating width of 7 mm was characterized by periodically moving at different relative sliding speeds with a given sliding displacement of 8 mm. Figures 3a and b show the output current and output voltage of the 2DW-WTNG at an average sliding speed of 0.025 m/s, 0.050 m/s, 0.075 m/s, 0.100 m/s, and 0.125 m/s, respectively. In the current curve and voltage curve, there is a full output peak in 320 ms in one-way moving and another output peak in 320 ms moving in the reverse direction at a sliding speed of 0.025 m/s. Within the same working time, an increase of the speed caused the increase of the output peak’s number from one at 0.025 m/s to five at 0.125 m/s. It was because a greater sliding speed shortened the time needed for one working cycle and further increased the number of the working cycles in the same working time. The current peak value was increased from 101 nA at 0.025 m/s to 415 nA at 0.125 m/s, which implied that an increase of the sliding speed could effectively increase the separation speed of the triboelectric charge and lead to a large output peak value. The voltage peak value was increased from 3.6 V at 0.025 m/s to 6.6 V at 0.125 m/s, which was resulted from the measuring electric circuit. The input resistances of the voltage measurement circuit and the 2DW-WTNG formed a RC electric circuit, and the leakage current on the 2DW-WTNG was reduced when increasing the sliding speed, resulting in continuous enhancement of the output voltage peak value. These results clearly demonstrate that the output peak value was closely related to the sliding speed. Apart from the sliding speed, sliding displacement is another factor which largely influenced the output performance of the 2DW-WTNG. Considering that most of the mechanical energy in human body motion is from movements of small amplitude, it is thus necessary to harvest the weak mechanical energy. To explore this aspect, the 2DW-WTNG was tested by working at a sliding displacement of 0.4 mm, 0.8 mm, 1.2 mm, 1.6 mm, and 2.0 mm with a fixed sliding speed of 0.1 m/s. The output current and voltage are shown in Figs. 3c and d. Its output peak value increased with the sliding displacement. At the shortest displacement of 0.4 mm, the output peak value reached 2.3 nA and 0.05 V, respectively, exhibiting its ability of scavenging mechanical energy from small motion. According to the working mechanism of the 2DW-WTNG in the horizontal-sliding mode, there was an alternating charge transfer when sliding over one grating. Thus, it is promising to further improve the output of the 2DW-WTNG under sliding mode by narrowing the width or diameter of the woven belts or woven fibers into smaller scale.

**Fig. 4:**
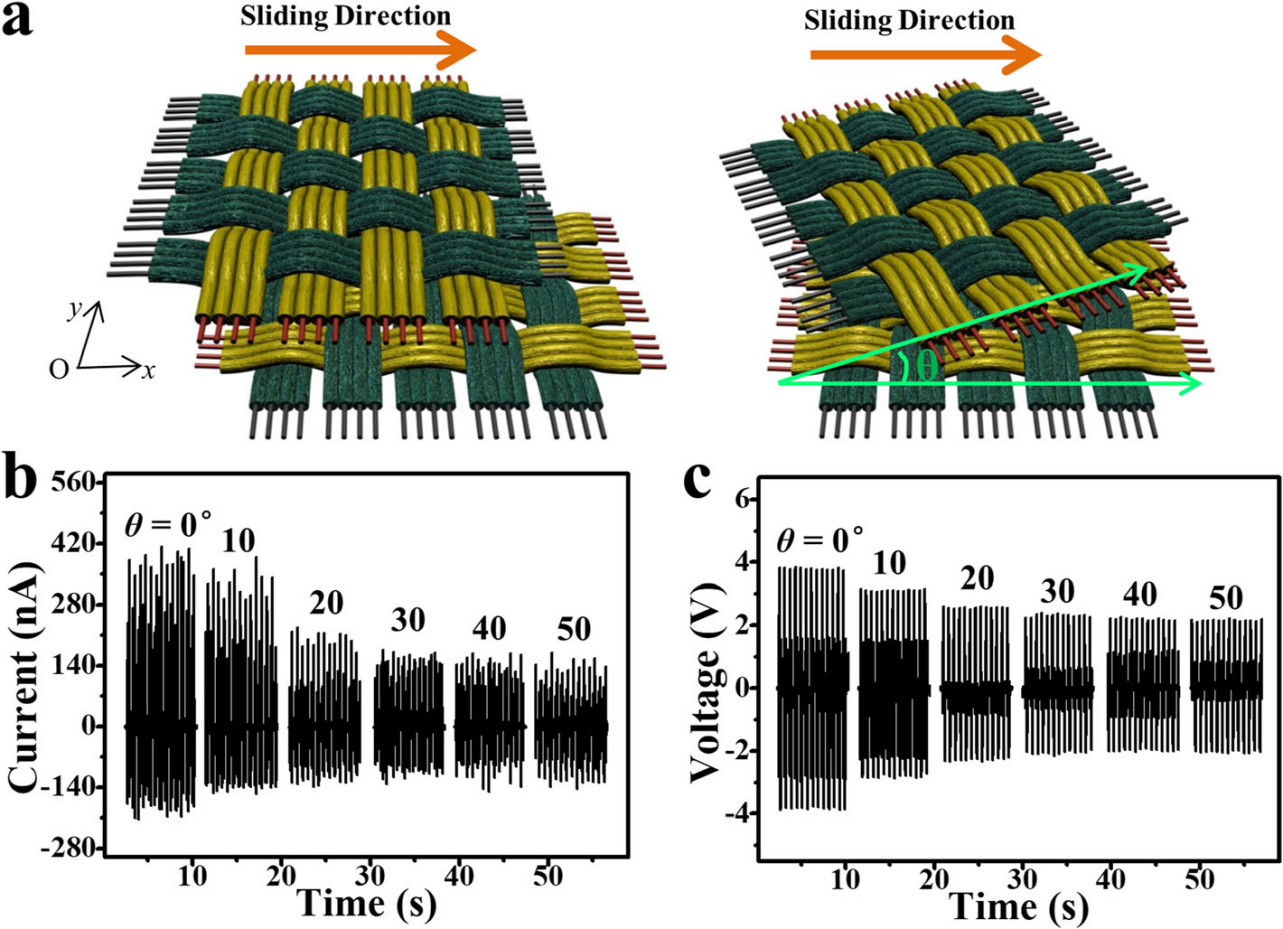
Adaptability of the 2DW-WTNG working along arbitrary in-plane directions. **a** Schematic diagram for the 2DW-WTNG working at different relative orientation. **b** Output current and **c** output voltage of the 2DW-WTNG at different relative orientation

Considering the complexity of human activities, the body motion energy may come from different directions. Therefore, a qualified WTNG should be able to harvest energy from body movements in different directions. In other words, a WTNG working in the planar-sliding mode is expected to work along arbitrary sliding directions. As shown in Fig. 4a, two parts of the 2DW-WTNG were placed face to face and the upper part could slide over the lower part along the *X*-axis. Keeping the moving direction, rotating the upper part resulted in an angle (*θ*) between the sliding direction and one side of the upper part. Here, the *θ* represented essentially the relative working orientation between the upper part and the lower part of the 2DW-WTNG, which required the 2DW-WTNG to be able to work at different relative working orientations. To elucidate this, the 2DW-WTNG was tested at a set of *θ* values (0°, 10°, 20°, 30°, 40°, and 50°) driven by the linear motor at a sliding speed of 0.10 m/s and a sliding displacement of 10 mm. Its output current and output voltage at different *θ* are shown in Figs. 4b and c. The device generated an output current of 134.45 nA and an output voltage of 2.23 V respectively at a relative working orientation of 50°. Meanwhile, due to the in-plane symmetry, the output current and output voltage at 40° were very close to those at 50°. Although the output current and output voltage decreased slightly as *θ* increases as a result of the decrease of the effective friction area caused by the mismatched gratings between the upper part and the lower part of the 2DW-WTNG, these experimental results strongly validated that the 2DW-WTNG could work normally at different working orientations. Benefited from the cylindrical composite fiber, its smooth surface made the sliding become continuously and reposefully, rather than the wobbling sliding in the narrow gratings with obviously raised edge made by lithographic process. Similarly, if an external motion along arbitrary in-plane direction was applied on the upper part of the 2DW-WTNG, it slid along the motion direction and rubbed with the lower part, and thus, the motion energy could be harvested and converted into electricity.

**Fig. 5:**
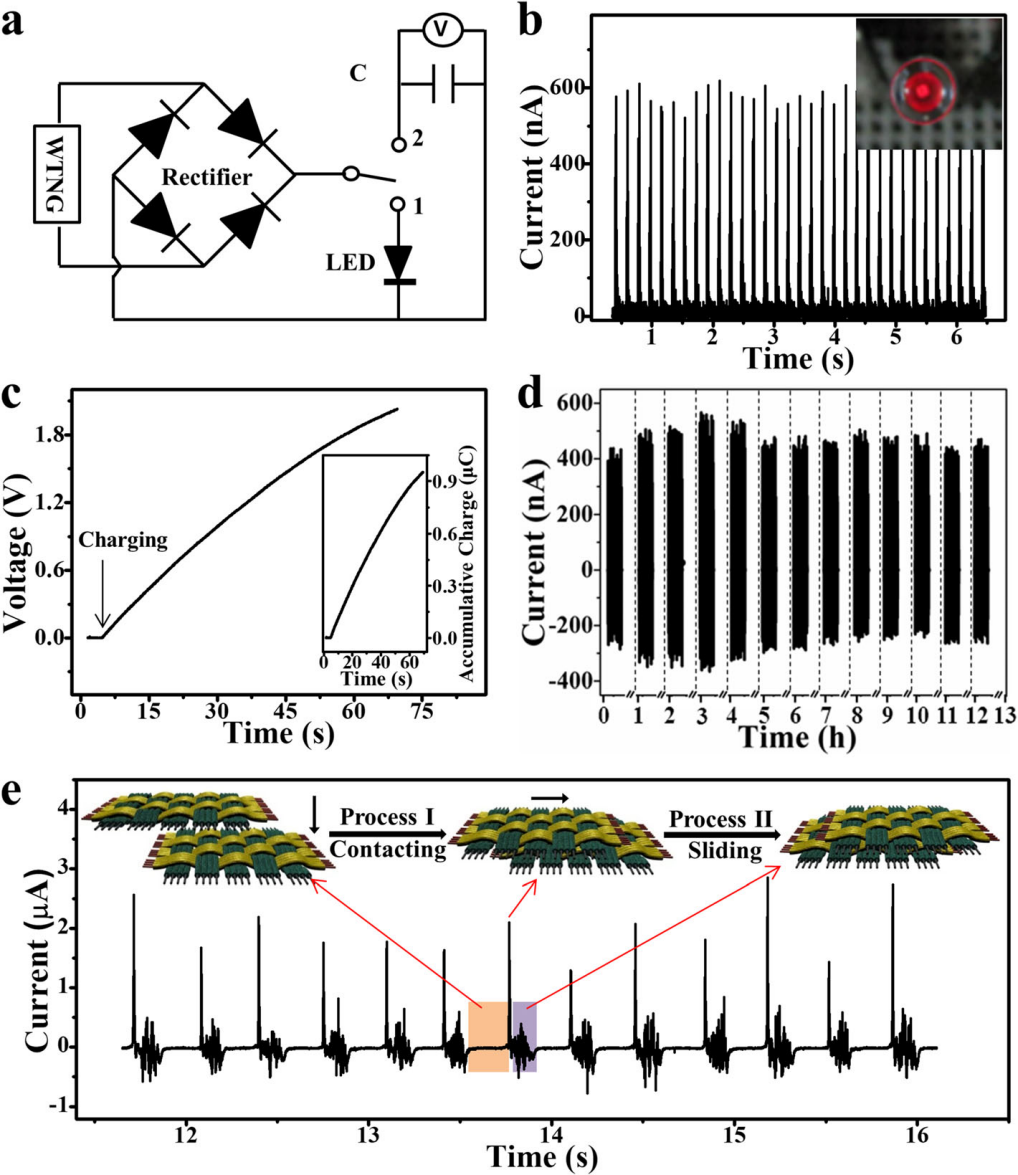
Application of the 2DW-WTNG. **a** Rectification circuit with two branch circuits at the output terminal. **b** Rectified output current signal. The inset is an optical image of a red LED powered by the rectified current signal. **c** Charging curve for a 0.47 μF commercial capacitor charged by the rectified current. The insert is the corresponding output charges stored in the capacitor. **d** Output current of the 2DW-WTNG working continuously for 12 h. **e** Output current of the 2DW-WTNG working in two different working modes, demonstrating the versatility of the 2DW-WTNG in scavenging human body motion energy. The working process is shown in the insert


As an energy harvester, the output of the 2DW-WTNG should be high enough for powering some electronic devices. As shown in Fig. 5a, the 2DW-WTNG was connected with a bridge rectifier and then connected with two branch circuits. With the bridge rectifier, the AC output of the 2DW-WTNG was transformed into direct current (DC) output. The rectified DC signal is shown in Fig. 5b. When connecting the bridge rectifier to the first branch circuit, the rectified DC signal was directly used to light up the red LED as shown in the inset and in Additional file 1: Video S1. When the bridge rectifier was connected to the second branch circuit, the electricity from the 2DW-WTNG charged a 0.47 μF commercial capacitor. The charging curve is shown in Fig. 5c and its corresponding charge amount stored in the capacitor is shown in the inset. The capacitor was charged to 1.84 V in 1 min, and the corresponding charge density reached 3.84 mC/m^2^. These two tests indicated that as an energy harvester, the 2DW-WTNG could not only be used as a convenient emergency power supply, but it could also fuel energy into a storage cell. Furthermore, the stability of the 2DW-WTNG is an essential factor to ensure its practical applications. Here, the 2DW-WTNG’s stability was tested by making it continuously works for 12 h at a sliding speed of 0.1 m/s and a sliding displacement of 8 mm. The current curves in 10 s for every hour are shown in Fig. 5d, and little change can be found in the output current value after 12-h continuous work, exhibiting highly stable power generation performance. Additionally, to demonstrate the versatility of the 2DW-WTNG in scavenging energy in various types, a group of electrical measurements was conducted on the 2DW-WTNG. As shown in the inset in Fig. 5e, the effective friction occurs as the upper part of the 2DW-WTNG firstly vertically contacted with the lower part of the 2DW-WTNG (process I), and then horizontally slid on the lower 2DW-WTNG (process II). During the measurement, the contacting-sliding-separating operation was repeated for several times, and its output current is shown in Fig. 5e. For the first vertically contacting friction process, there was a high but narrow output peak, and then for the following horizontally sliding friction process, there was a low but wide peak. At the last vertically separating process for next measuring cycle, a high but narrow output peak should appear but was missing. This can be attributed to two reasons. One is that the upper part of the 2DW-WTNG has slid out the opposite lower part of the 2DW-WTNG, and the electric potential was almost balanced. The other one is that the last vertically separating process was rather slow, so the electric potential quickly reached equilibrium in the air. The synthetical analysis confirmed that these two kinds of output current peaks were consistent with the characteristics of 2DW-WTNG’s two working modes, displaying its strong adaptability to scavenge energy from vertical positive pressure and horizontal tangential force in human body motions.

Additional file 1: **Video S1.** Video of instantly lighting a red LED. (AVI 1334 kb)


## Conclusions

In summary, a new WTNG with 2D woven structure as a wearable power source was developed through an easily scalable approach. This 2DW-WTNG has demonstrated its good capability of converting the mechanical energy into electricity and generated a current density up to 2.73 mA/m^2^. It could instantaneously power a commercial red LED without the need for an energy storage process. It can also be used to charge a 0.47 μF capacitor to 1.84 V in 1 min and the charge density reached 3.84 mC/m^2^ in 1 min. Benefited from the robustness of the core-shell structured fiber and the woven structure, the 2DW-WTNG could work in arbitrary sliding directions. Furthermore, the 2DW-WTNG was applied to harvest mechanical energy with different forms and worked continuously for 12 h with steady output. The remarkable performance, flexibility, maneuverability, and robustness enabled the 2DW-WTNG to harvest the mechanical energy from human body motion and to power low-power electronic products. Most importantly, this work provides a designed model for massive production of fiber-based wearable generator, which will greatly promote the development of personal electronic devices.

## Data Availability

The data and the analysis in the current work are available from the corresponding authors on reasonable request.

## Abbreviations

2DW-WTNG: 2D woven wearable triboelectric nanogenerator
AC: Alternating current
DC: Direct current
LED: Light-emitting diode
SEM: Scanning electron microscope
WTNG: Wearable triboelectric nanogenerator
